# Hydrophobic Amino Acids as Universal Elements of Protein-Induced DNA Structure Deformation

**DOI:** 10.3390/ijms21113986

**Published:** 2020-06-02

**Authors:** Kateřina Faltejsková, David Jakubec, Jiří Vondrášek

**Affiliations:** 1 Institute of Organic Chemistry and Biochemistry, Czech Academy of Sciences, Flemingovo náměstí 542/2, 166 10 Prague 6, Czech Republic; katerina.faltejskova@seznam.cz; 2 Department of Cell Biology, Faculty of Science, Charles University, Viničná 7, 128 00 Prague 2, Czech Republic; 3 Department of Physical and Macromolecular Chemistry, Faculty of Science, Charles University, Hlavova 8, 128 40 Prague 2, Czech Republic

**Keywords:** protein–DNA interaction, DNA shape, minor groove, specific recognition, hydrophobic, indirect readout

## Abstract

Interaction with the DNA minor groove is a significant contributor to specific sequence recognition in selected families of DNA-binding proteins. Based on a statistical analysis of 3D structures of protein–DNA complexes, we propose that distortion of the DNA minor groove resulting from interactions with hydrophobic amino acid residues is a universal element of protein–DNA recognition. We provide evidence to support this by associating each DNA minor groove-binding amino acid residue with the local dimensions of the DNA double helix using a novel algorithm. The widened DNA minor grooves are associated with high GC content. However, some AT-rich sequences contacted by hydrophobic amino acids (e.g., phenylalanine) display extreme values of minor groove width as well. For a number of hydrophobic amino acids, distinct secondary structure preferences could be identified for residues interacting with the widened DNA minor groove. These results hold even after discarding the most populous families of minor groove-binding proteins.

## 1. Introduction

Vital cellular processes, such as DNA replication, transcription, and gene expression are both regulated and executed by proteins. Specific recognition of DNA sequences is essential for these processes. The number of experimentally determined three-dimensional (3D) structures of protein–DNA complexes is increasing, providing an opportunity to uncover the underlying mechanisms of specific DNA sequence and structure readout.

The mechanisms of specific recognition of DNA sequences can be divided into direct (base) readout and indirect (shape) readout. During direct readout, a pattern of functional groups belonging to DNA bases (hydrogen bond donor and acceptor groups, thymine methyl group) is matched by complementary groups of the protein [1,2]. Direct readout achieves high specificity in the DNA major groove, as every base pair provides a unique pattern of hydrogen bond donor and acceptor groups. In the DNA minor groove, however, certain base pairs expose patterns of hydrogen bond donor and acceptor groups that are indistinguishable from each other. In particular, distinctions between the GC and CG base pairs and between the AT and TA base pairs are impossible [2,3].

Indirect readout plays a key role in achieving specificity in the DNA minor groove [4]. This group of recognition mechanisms includes all forms of recognizing the DNA structure and its variations [1,2]. Several of these variations are correlated with DNA sequence. For example, AT-rich regions are generally more flexible, and multiple structural variations are associated with them [5]. Specifically, decreased stacking interactions in TpA steps can introduce kinks in the helix [6]. TATA-box sequences are prime examples of sequences rich in TpA steps. They adopt a “tilted A-DNA (TA-DNA)” structure [2] that is specifically bound by the TATA-binding protein (TBP) using indirect readout. The minor groove of this sequence is wide open even without the protein factor present [7].

AT-rich sequences without TpA steps tend to display a narrow minor groove [8,9]. These sequences and other helices with narrow minor grooves often interact with arginine residues [10]. This phenomenon can be explained by the enhanced negative electrostatic potential in the narrow minor groove that is recognized specifically by positively charged arginine residues. In contrast, GC-rich sequences tend to have wider minor grooves [11]. The width of the minor groove is a widely used descriptor of the DNA structure and can be recognized during indirect readout. Hancock et al. observed that increase in the minor groove width can prevent binding of Fis protein [12]. The DNA minor groove width is influenced by other DNA features as well, such as the sugar-phosphate backbone conformation [13].

Mechanisms belonging to both the direct and indirect readout categories participate in the DNA recognition, although their relative contributions depend on the nature of the protein–DNA complex formed [1,14]. As the structural features of DNA are determined by the specific DNA sequence to a lesser extent than the pattern of hydrogen donor and acceptor groups [15], the mechanisms of indirect readout are more variable. This is one of the reasons that indirect readout mechanisms are less understood than those of direct readout [1,4,16].

Arginine enrichment in narrow minor grooves is not the only example of correlation between DNA shape and the protein–DNA interface composition. Hydrophobic amino acids may contact the DNA helix, mostly in the vicinity of nucleotides with a C3’-endo sugar conformation, which is associated with A-DNA conformation [17]. Another form of contacting the minor groove by a hydrophobic amino acid residue is intercalation of the residue into the DNA helix. During the binding of a TATA-box by the TBP, two phenylalanine residues intercalate into the DNA to stabilize the kink in the DNA double helix [16,18]. The intercalation of phenylalanine, leucine, or other hydrophobic residues into the DNA structure has been observed for several proteins that bind the DNA minor groove, including mitochondrial transcription factor A, male sex-determining factor SRY and bacterial catabolite control protein A. In these cases, the importance of the intercalated residues for modifying DNA structure was verified by molecular dynamics simulations of protein–DNA complexes in which these residues were replaced [18].

The mechanism of DNA recognition by a protein is reflected by classifications of proteins into families based on their ability to read the DNA sequence as well as the DNA shape [16,19]. Evidence suggests that a particular set of DNA structural features can be recognized specifically by a family of transcription factors and the values of DNA structural descriptors (e.g., propeller twist, helical twist, roll, and minor groove width) can help discern DNA-binding sites of different transcription factor families [19]. This is echoed in the binding sites of the transcription factors, which tend to have conserved structure if not conserved sequence [20].

**Figure 1:**
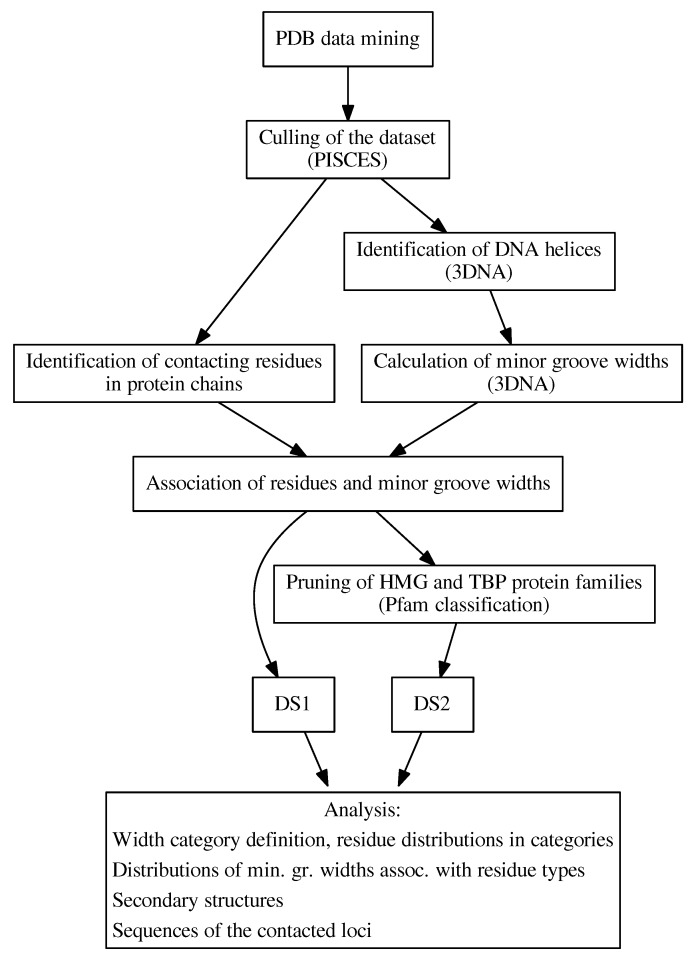
An overview of the workflow and analyses performed in this work. Individual steps are described in detail in Section 3.

In this study, we comprehensively analyzed the inter-molecular interfaces between DNA minor grooves and proteins in 3D structures of protein–DNA complexes obtained from the Protein Data Bank (PDB) [21,22,23] in order to understand the relationship between the amino acid composition of the protein interface and the local properties of the DNA, focusing mainly on the structural features of the DNA helix (Figure 1).

We developed an algorithm to detect DNA minor groove-contacting amino acid residues, and the identified residues were associated with the local groove width. Our findings revealed a link between the presence of hydrophobic amino acid residues at the protein–DNA interface and increased minor groove width. We further determined that this phenomenon is not protein family-specific by repeating the analysis with known large minor groove-binding families excluded. In addition, we described the preferences of amino acid residues associated with specific DNA minor groove width categories to form various secondary structures, and showed that distinct preferences exist in several cases. Further study of the relationship between the minor groove dimensions and the DNA sequence showed that widening of the DNA minor groove is linked with the presence of GC-rich sequences.

## 2. Results and Discussion

**Figure 2:**
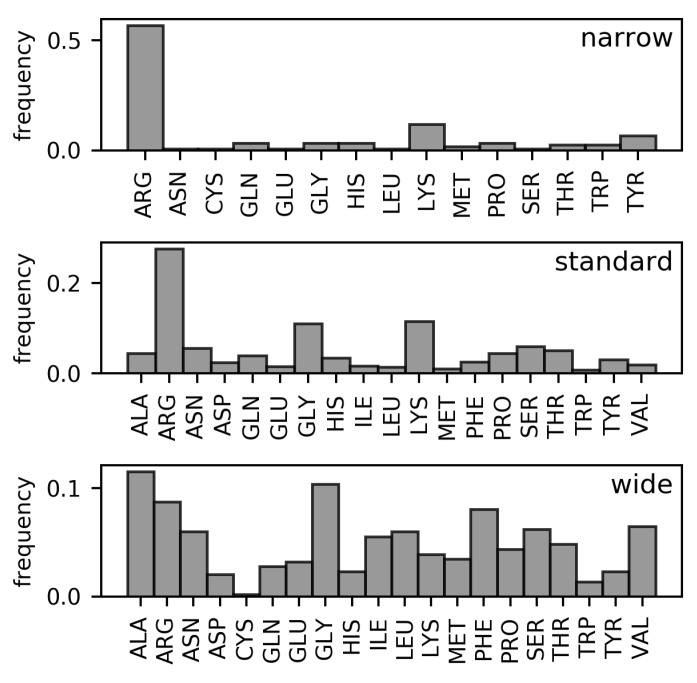
Frequencies of amino acids contacting narrow, standard, and wide DNA minor grooves (DS1 dataset).

**Figure 3:**
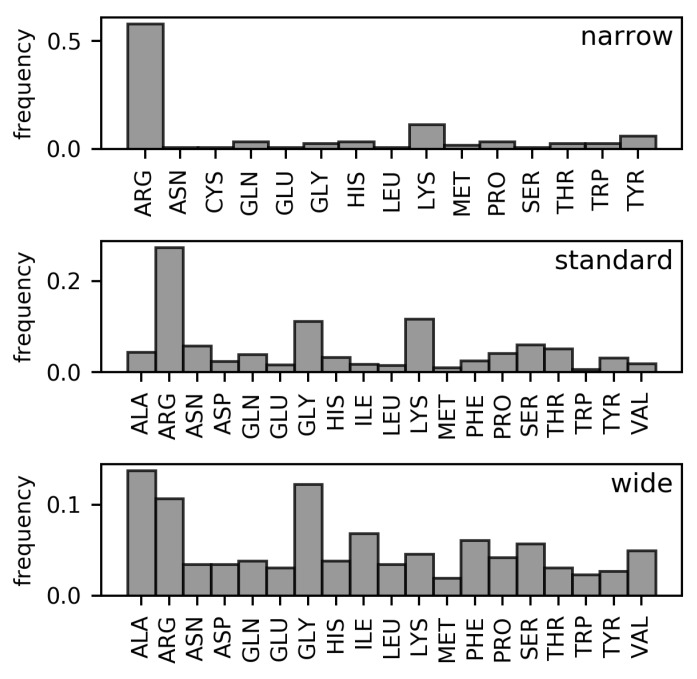
Frequencies of amino acids contacting narrow, standard, and wide DNA minor grooves (DS2 dataset).

Analyses of the frequencies of amino acids found in the three DNA minor groove width categories in the DS1 and DS2 datasets (These datasets are described in detail in the Materials and Methods section. Both are non-redundant datasets of amino acid–dinucleotide step pairs; in DS2, well-known families of minor groove-binding proteins have been further removed). (Figure 2 and Figure 3, respectively) have revealed the following differences in residue distributions.

Arginine is the most frequent DNA-contacting residue in the narrow and standard minor grooves (56.8% and 27.6% of narrow and standard minor groove-contacting residues, respectively). The enrichment of arginine residues in the narrow minor grooves was examined and explained in the work of Rohs et al. [10]. However, their supremacy is lost in the wide minor grooves (8.8% of wide minor groove-contacting residues). In this category, no single type of residue clearly dominates. Instead, arginine residues tie with alanine (11.5%) and glycine (10.4%) in terms of frequency amongst the wide minor groove-contacting amino acids. Frequencies of other hydrophobic amino acids (leucine, isoleucine, valine, phenylalanine) are higher in this category compared with narrow and standard minor grooves. In sum, the total portion of hydrophobic amino acid residues in contact with wide minor grooves (57.3% counting the mentioned amino acids, plus proline, tryptophan, and methionine) is comparable to the portion of arginine residues contacting the narrow minor grooves.

In DS1, the frequencies of leucine and phenylalanine residues in contact with the wide minor grooves are comparable to the frequencies of alanine and glycine. However, the proportion of leucine and phenylalanine residues in contact with the wide minor groove is reduced in the DS2 dataset (from 6.0% to 3.5% for leucine and from 8.1% to 6.1% for phenylalanine). However, hydrophobic amino acid residues still predominate in this width category in the DS2 with total portion of 55.7%.

The overall differences between the distributions of amino acid residues in contact with narrow, standard, and wide minor grooves were quantified using relative entropy (Table 1). From the relative entropy values, we can infer that the distribution of amino acids in contact with the standard minor grooves cannot describe the narrow and wide minor groove distributions, as approximately 0.5 bit of information would be lost in both cases. The difference between the distributions of amino acids in contact with the wide and narrow minor grooves is even greater and indicates completely dissimilar compositions of the respective minor groove-binding motifs.

An increased presence of hydrophobic amino acid residues was previously observed in the minor grooves involving a nucleotide in the C3’-endo sugar pucker, which correlates with the A-DNA conformation [17]. As the minor groove width in an ideal A-DNA conformation reaches 18.5 Å (value taken from a model provided by the 3DNA program), it would be placed into the wide minor groove category, in agreement with the propensities of hydrophobic amino acids described in this study. However, in our dataset, sugar puckerings in the wide minor grooves vary, and the C3’-endo conformation is observed in only 11% of cases. Interestingly, in more than 80% of dinucleotide steps, there is at least one nucleotide with a sugar conformation other than C3’-endo or C2’-endo.

Hydrophobic amino acid residues were also previously observed in more distorted DNA helices, such as the TATA-box bound by the TBP. While these distorted helices differ from the ideal A-DNA, they display a wide minor groove as well. In these structures, certain residues (namely, leucine, methionine, phenylalanine, and valine) can have a stabilizing role [16,18].

**Figure 4:**
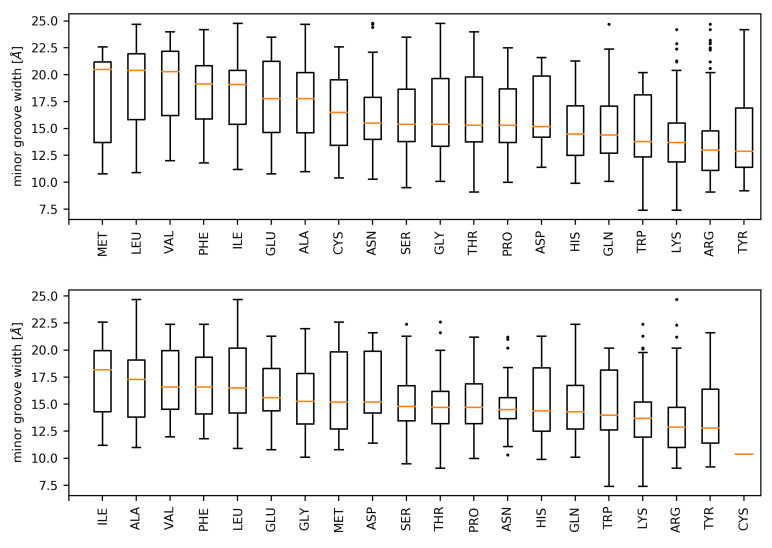
Distributions of DNA minor groove widths associated with the dinucleotides bound by the respective amino acids (top: DS1, bottom: DS2). Amino acids are sorted in descending order according to the associated median minor groove width. Whiskers of the boxplots denote the first and last 25% of the distributions. Maximal length of a whisker was set to 1.5 times the interquartile range. Any observations belonging to the whisker range that is further from the first/third quartile than this threshold is denoted as an outlier and marked as a dot in the plots.

We next investigated the distributions of minor groove widths associated with every type of contacting residue (Figure 4). The highest values of median minor groove width were associated with hydrophobic amino acids (isoleucine, leucine, alanine, phenylalanine, and valine) in both datasets. On the other hand, another hydrophobic amino acid, tyrosine, was associated with the lowest median minor groove width in both DS1 and DS2.

We identified several differences between the DS1 and DS2 datasets in this regard. In the DS1 dataset, more than half of methionine residues contact an extremely wide minor groove (width higher than 20 Å). However, many of these residues are excluded from the DS2 dataset, which results in an approximately 5 Å lower median minor groove width. The methionine residues excluded from the DS1 dataset are classified in the TBP family.

As shown by the one-sided Mann–Whitney *U* test (Supplementary Table S1), on a confidence level of 95%, the median minor groove width in both DS1 and DS2 datasets is significantly higher when the dinucleotide in question is in contact with alanine, isoleucine, leucine, glycine, phenylalanine, valine, and glutamic acid. Out of these amino acids, only the glutamic acid cannot be categorized as hydrophobic. This phenomenon is discussed further in more detail.

The Mann–Whitney *U* test shows that the preference to contact minor grooves with increased width persists for the selected amino acids even when the residues from the most frequent protein families are excluded from the analysis. Despite the propensities of leucine, phenylalanine, or valine being lowered in the DS2 in comparison with DS1, the distributions of minor groove widths associated with said amino acids are still significantly shifted. It is thus suggested that the phenomenon of the specified amino acids recognizing wide minor grooves is not specific to the TBP and HMG protein families, and may constitute a universal mechanism of specific DNA binding. The fact that no other protein families were significantly enriched in either dataset also hints that hydrophobic amino acid enrichment in the wide minor groove-binding motifs is not a protein family-specific readout feature similar to those described in the work of Yang et al. [19].

As a single dinucleotide step can be contacted by multiple amino acid residues, its width value can propagate into all the respective associated minor groove width distributions. For certain amino acid types, a glycine residue was observed as the preferred partner; for example, 75% of dinucleotide steps associated with an aspartate residue were contacted by a glycine residue as well. In addition to aspartate, this phenomenon was observed for dinucleotide steps associated with histidine residues (70% of histidine-contacted steps are also contacted by glycine), isoleucine residues (58% of isoleucine-contacted steps are also contacted by glycine) and tyrosine residues (53% of tyrosine-contacted steps are contacted by glycine as well). While the distributions of minor groove widths associated with these residues could be influenced by the presence of glycine, we consider it unlikely, as said distributions are dissimilar (as seen in Figure 4). Moreover, the fraction of dinucleotide steps contacted by a glycine residue as well as by aspartate, histidine, isoleucine, or tyrosine never exceeds 13% of all glycine-contacted steps.

### 2.1. Secondary Structures of Amino Acids Contacting the Minor Groove

We assessed the secondary structure preferences of hydrophobic amino acids in standard and wide minor groove width categories (Table 2). As these types of residues are rarely in contact with a narrow minor groove, no secondary structure preference is available for this category.

We identified clear secondary structure preferences for certain amino acids. Glycine, isoleucine, and valine residues were most frequently found in a β-strand conformation when binding the wide minor grooves, while this conformation is rarely seen in the standard minor grooves. For valine residues, this secondary structure preference appears to be restricted to the TBP and HMG protein families, as is apparent from the decreased numbers of observations in the pruned dataset.

On the other hand, phenylalanine residues were mostly found in the α-helix conformation in wide minor grooves. This conformation appears to be specific to the HMG protein family. The α-helix conformation is also preferred by tyrosine residues found in the wide minor grooves. However, tyrosine residues were more frequently found in the standard minor grooves, prevalently in a polyproline-II conformation.

The preferences were more obscure for leucine and alanine. For both amino acids, the numbers of residues in an α-helix conformation were increased in the wide minor grooves compared with the standard minor grooves, but the differences are not substantial. Approximately half of alanine and leucine residues in the α-helix conformation in contact with the wide minor groove originate from the HMG family.

Occurences of residues in β-strand conformation were sparse in standard minor grooves but more frequent in wide minor grooves. Two DNA-binding motifs have been recognized that are composed of residues in the β-strand conformations: β-sheet and β-hairpin [24]. As the β-sheet DNA-binding motif is characteristic for the TBP family [25], preferences for the β-strand conformation are generally lowered for most amino acid types in the DS2 dataset. In spite of this, the portion of isoleucine residues in the β-strand conformation was almost the same in both datasets.

In wide minor grooves, hydrogen bonds between protein and DNA were rarely observed. The hydrophobic amino acid residues enriched in the wide minor grooves form no such interactions. The only exception were 5 isoleucine residues that were observed forming a hydrogen bond with a DNA base. All of these residues contact wide minor grooves. Instead of hydrogen bonds, van der Waals interactions were dominant. The complete list of amino acid propensities to create hydrogen bond or van der Waals interactions can be found in Supplementary Figure S1; the populations of hydrogen bonds between the amino acid backbone and DNA bases are shown in Supplementary Table S2. It can be seen that glycine residues are the only ones which form significant populations of the backbone–base interactions (in the standard minor grooves).

Tyrosine residues in the standard minor grooves frequently form hydrogen bonds with the deoxyribose in the DNA sugar-phosphate backbone. This interaction motif is almost exclusively linked with protein backbone dihedral angles characteristic for a polyproline-II helix. Hydrogen bonds between tyrosine side chain and the nucleobases or the phosphate groups of the DNA were also observed; however, these interaction motifs were observed for other tyrosine secondary structure conformations as well.

### 2.2. Properties of DNA Helices Contacted by Different Amino Acids

**Figure 5:**
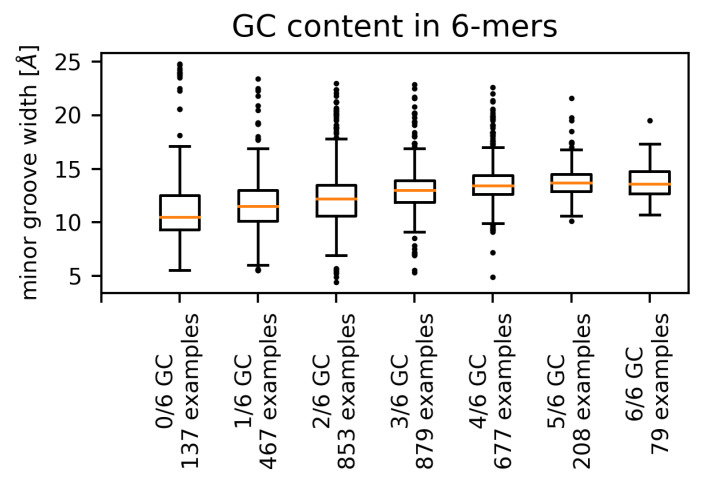
Distributions of DNA minor groove widths corresponding to hexamers with different values of GC content (complete non-redundant dataset). The numbers of examples refer to the number of hexamers with said GC content.

We observed a positive correlation between the GC content of the hexamers and the (mean) minor groove width for the pooled set of all contacts (Figure 5), as well as for single amino acids. This phenomenon appears to be independent of the contacting amino acid, as higher GC content has been shown to correlate with wider minor groove [10,14,26].

For a number of amino acids (phenylalanine, glycine, isoleucine, leucine, and valine), the distribution of minor groove widths contains some extremely high values corresponding to AT-only sequences. The AT-rich sequences containing extremely wide minor grooves are not confined to regions contacted by hydrophobic acids. We identified such examples for threonine, proline, glutamate, and tyrosine residues, although in these cases a hydrophobic amino acid can be found in contact with the same hexamer.

**Figure 6:**
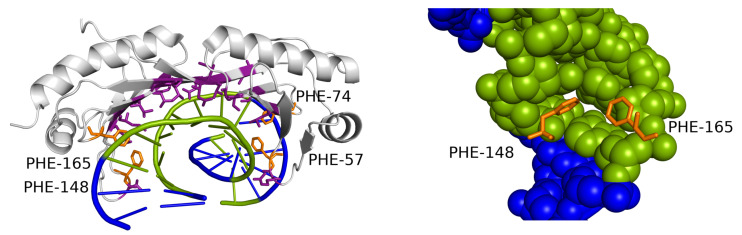
TBP DNA-binding motif interacting with the extremely widened minor groove. The contacted DNA sequence consists only of AT base pairs (green). Two phenylalanine residues (orange) intercalate into the helix on one or both edges of the motif (detail on the right). A similar DNA-contacting motif appears in the dataset also in 4ROC and 1YTB structures, both containing similar extreme deformations of the minor groove in AT-rich sequences.

The range (variance) of possible widths is the lowest for GC-only sequences and the highest for the AT-only sequences. The fact that AT-rich sequences have been observed to form both extremely narrow [10] and extremely wide minor grooves reflects the general greater flexibility of the AT-rich sequences [5] in comparison with mixed or GC-rich sequences. Most of the extreme values of minor groove width corresponding to AT-rich sequences originated in four PDB structures. Three of these structures, 1QNA, 1YTB and 4ROC (TBP/TATA-box complex in *Arabidopsis thaliana*, yeast and human), contact the minor groove via a β-sheet with two phenylalanine residues on the edge of the interface. These phenylalanine residues intercalate between two corresponding AT dinucleotide steps (Figure 6) [18].

**Figure 7:**
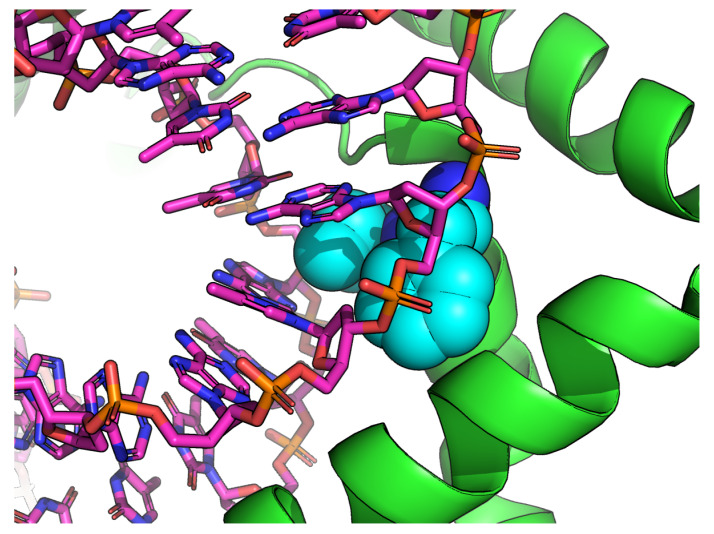
The non-intercalating interaction in the structure 5JH0. Bulky, hydrophobic residues (Phe, Ile) are shown in teal. Visualized using PyMOL-1.7.2.1 [27].

The fourth structure (mitochondrial DNA packaging protein Abf2p in complex with DNA, PDB ID 5JH0) contains an AT-only sequence contacted by hydrophobic amino acids (Figure 7).

Here, the binding motif consists mainly of a α-helix secondary structure. Intercalation of hydrophobic residues into the DNA helix is thus not the only way of stabilizing the wide minor groove, although this type of interaction seems to result in the most extreme values of minor groove width.

Some amino acids exhibited preferences for certain sequences of the contacted dinucleotide step (Supplementary Figures S2 and S3). For example, 60% of leucine residues were in contact with dinucleotide steps consisting only of AT and TA base pairs and half of these (30% of all leucine residues) were in contact with ApA/TpT dinucleotide steps. Similarly, more than 40% of all dinucleotide steps contacted by isoleucine comprise only of AT and TA base pairs, approximately half of it (22% of all isoleucine residues) being ApA/TpT dinucleotide steps. Valine and alanine residues were also found to contact dinucleotide steps consisting only of adenine and thymine (40% of contacted dinucleotides display such a composition). However, these two amino acids do not seem to have any preference for a particular sequence.

**Figure 8:**
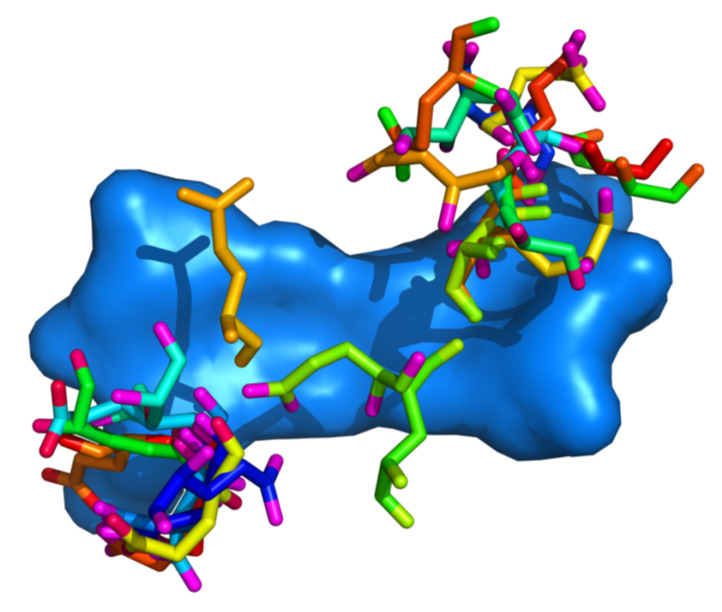
Positioning of glutamic acid residues with regards to the DNA dinucleotide. Residue–dinucleotide steps were superimposed using the Kabsch algorithm. All dinucleotide steps are represented by the step with minimal RMSD from all other steps.

A preference for dinucleotide steps that comprise only of cytosine and guanine was observed for aspartic acid (48% of all aspartic acid residues) and glutamic acid (45% of all glutamic acid residues). The CpC/GpG dinucleotide steps were most favoured, as 33% of aspartic acid residues and 28% of glutamic acid residues were observed in contact with these dinucleotides. The preference for GC-rich sequences displayed by glutamic acid could help explain the significantly higher minor groove width values observed in some cases. The glutamic acid residues do not seem to contact the nucleobases directly. Instead, they tend to cluster in proximity to the DNA backbone (Figure 8). The interaction between phosphate groups in the DNA backbone and glutamate residues has been described as weak [24].

It remains unclear whether the presence of hydrophobic amino acids at the interface causes minor groove widening or whether the wide minor grooves are recognized by DNA-binding motifs rich in hydrophobic amino acids. Various studies have provided evidence that the minor groove shape and width influence protein binding [7,12,28,29], suggesting the latter to be true. However, according to a molecular simulation, replacement of phenylalanine, valine, and methionine residues that intercalate into the DNA helix affects the DNA shape in a protein–DNA complex. Replacing certain phenylalanine residues in the TBP DNA-binding motif is an example of this phenomenon [18], supporting the first possibility. An interplay of both options should also not be dismissed, as in the case of TBP, the TATA-box is deformed on its own [2,7] and can facilitate the specific recognition as well.

## 3. Materials and Methods

All 3D structures containing protein and DNA chains were downloaded from the RCSB PDB database on 30 August 2018. A total of 4783 structures were acquired. As DNA is mostly present in the cell in the form of a double helix, our study of DNA recognition was limited to this conformation. To identify and analyze all the DNA helices, the 3DNA program (v2.3.2-2017dec26) was employed [30]. The search was limited to DNA helices containing no HETATM records. All DNA helices were validated to consist only of the standard deoxyribonucleotides (dA, dC, dG, dT) and to have complete information on the non-hydrogen atom positions for each of them. DNA chains that did not form a double helix were not considered in the following analysis.

Structures containing a validated DNA helix were submitted to the PISCES server [31] to obtain a non-redundant set of protein chains. This set was filtered to contain only the structures obtained using X-ray diffraction and resolved to a resolution of 3.5 Å or better. In this step, the dataset was also refined to be composed only of protein chains with a 90% maximal mutual sequence identity. This final non-redundant dataset consisted of a total of 976 protein chains found in 857 PDB structures.

### 3.1. Identification of Minor Groove-Binding Residues

To define an amino acid residue in contact with a DNA groove, two reference atoms were defined for each nucleobase, one residing in the major and the other in the minor groove. Each reference atom sits approximately in the center of the respective groove. The reference atoms for the minor and major grooves, respectively, were defined as N3 and N6 for adenine, N3 and O6 for guanine, O2 and O4 for thymine, and O2 and N4 for cytosine.

For every amino acid residue in the non-redundant dataset, the nearest reference atom in the corresponding DNA helix was found. The distance between a DNA reference atom and an amino acid residue was calculated as the shortest Euclidean distance between the reference atom and any non-hydrogen atom of the amino acid residue. To acquire a set of minor groove-contacting amino acid residues, the residues for which the nearest reference atom belonged to the minor groove were collected. If the distance between the reference atom and the amino acid residue was less than 6.0 Å, the residue was labeled as minor groove-contacting and included in the dataset.

Fourteen amino acid residues assigned to the DNA minor groove using this algorithm were found to contact the major groove by visual analysis. These amino acid residues were manually removed from the dataset.

### 3.2. Characterization of DNA Minor Groove Dimensions

To each minor groove-contacting amino acid residue, we further assigned a dinucleotide step consisting of the nearest contacted nucleotide and the closer of the two adjacent nucleotides. Distances were calculated between the reference atoms of the nucleotides and the amino acid in question as described above.

The minor groove width for each dinucleotide step was retrieved from the output of the 3DNA analysis. Using this procedure, we obtained a dataset (DS1) comprising 1293 minor groove-contacting amino acid residues, each associated with a dinucleotide step and local minor groove width. The numbers of residues of each amino acid type in the DS1 dataset are listed in Table 3. Every amino acid residue in the DS1 is linked with one dinucleotide step. However, a single dinucleotide step can be contacted by multiple amino acid residues simultaneously.

**Figure 9:**
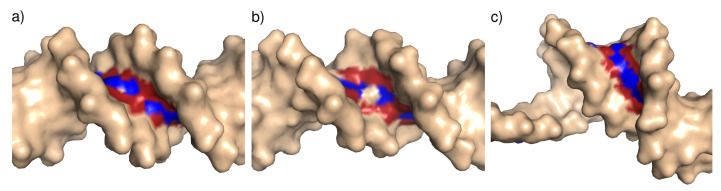
Illustration of the minor groove width categories: (**a**) Narrow minor groove (T4 Endonuclease VII H43N mutant in complex with heteroduplex DNA, PDB ID 2QNF); (**b**) standard minor groove (human Pax-6 paired domain–DNA complex, PDB ID 6PAX); (**c**) wide minor groove, (topoisomerase II alpha bound to DNA, PDB ID 4FM9). In this case, the wide minor groove is associated with bending of the DNA structure. The minor groove nitrogen and oxygen atoms are colored blue and red, respectively.

Three minor groove dimension categories were defined based on the groove width corresponding to a particular dinucleotide step: Narrow (≤11.0 Å), standard (between 11 and 17 Å), and wide (≥17 Å). The values of 11 and 17 Å correspond approximately to the 15th and 85th percentiles of minor groove width distribution; the definition of the narrow minor groove category is also similar to that used by Rohs et al. [10]. The three width categories are illustrated in Figure 9.

### 3.3. Identification of Protein Families

The minor groove-contacting amino acid residues were assigned to protein families using the InterProScan program [32]. The distribution of protein families in the dataset according to the Pfam [33] database was examined and the most abundant protein families in each width category were identified. Next we compiled a list of Pfam families that have a significant presence in the wide minor groove-binding category and are known from literature to bind distorted DNA structures. Among such families, the most abundant was the High mobility group protein family (PF00505), containing 16% of residues in the specified category. The second most common Pfam family of this type, TATA-box binding proteins (PF00352), contained 13% of the residues. No other Pfam family had an abundance exceeding 10% of residues. A second dataset of minor groove-contacting amino acid residues was generated by excluding all residues belonging to these two abundant protein families. A total of 1103 amino acid residue–dinucleotide step pairs were included in this pruned dataset, denoted DS2 (see Table 3).

### 3.4. Amino Acid Distributions in the DNA Minor Groove Width Categories

To examine whether specific amino acid residues are significantly associated with particular DNA minor groove width categories, two statistical tests were employed. First, the distribution of the DNA minor groove widths found in a specific set of dinucleotide steps (e.g., those contacted by a particular amino acid residue) was compared to another distribution using a one-sided Mann–Whitney *U* test [34]. For this test, the amino acids were separated into a hydrophobic group (Leu, Ala, Ile, Val, Gly, Phe, Trp, Tyr, Met) and a non-hydrophobic group (Asp, His, Glu, Gln, Asn, Thr, Ser, Lys, Cys, Pro). The minor groove widths associated with dinucleotides bound by arginine residues were excluded from the datasets used in this test because their numerousity and low minor groove width skewed the respective distributions. For each of the hydrophobic amino acids, the distribution of minor groove widths associated with the dinucleotide steps contacted by the residue was compared to the distribution of widths corresponding to dinucleotides contacted by all amino acids from the non-hydrophobic group. For each non-hydrophobic amino acid, the corresponding width distribution was compared to that of all other non-hydrophobic amino acids. In these tests, the null hypothesis states that the median groove widths found in the respective sets are equal; the alternative hypothesis states that the median of the first distribution is greater than the median of the other.

In addition, pairwise differences in the amino acid composition of the protein–DNA interfaces corresponding to the three minor groove width categories were measured by calculating the relative entropies of the respective amino acid distributions.

### 3.5. Analysis of DNA Sequence Features

The frequencies of contacted dinucleotide sequences were calculated for each amino acid, and the frequencies of amino acids binding each dinucleotide sequence were obtained. To assess whether any of the amino acid–dinucleotide sequence pairs are associated with particular protein families, every amino acid residue was linked with a Pfam family (if the residue belonged to a protein family) as described, and the frequencies of protein families binding individual dinucleoride sequences were calculated.

To probe the non-local properties of the DNA helices, a protein–DNA interface was defined as a continuous sequence of dinucleotide steps contacted by amino acid residues, with breaks of up to two consecutive non-contacted dinucleotide steps allowed. The interfaces were then examined using a 6-nucleotide sliding window. A width was assigned to every such hexamer equal to the value corresponding to the central dinucleotide step. All amino acid residues contacting any of the dinucleotides contained in the hexamer were labeled as hexamer-contacting. This approach was chosen to reflect the method of measuring the minor groove width by the 3DNA program [35].

The GC content (% of GC pairs) was calculated for every hexamer. The distribution of the respective hexamer widths was determined for each of the seven possible values of GC content both by considering all contacting amino acid residues together, as well as each amino acid separately. If an amino acid was present among the hexamer-contacting amino acid residues, the width of this hexamer was included in the distribution corresponding to the amino acid.

### 3.6. Properties of the Minor Groove-Contacting Amino Acid Residues

To study the protein secondary structures containing the DNA-contacting amino acid residues, all 3D structures of protein–DNA complexes were analyzed using the DSSP program (v2.2.1) [36]. The frequencies of individual secondary structures were then calculated for every amino acid. Ramachandran plots were prepared for all combinations of DNA minor groove width categories and amino acids using the backbone dihedral angle values determined by DSSP. Hydrogen bonds and other interaction modes between the DNA and amino acids were identified using the SNAP program (v2.42) from the 3DNA suite using its default settings. This information was plotted into the Ramachandran plots to see whether there is a link between an interaction mode and the protein secondary structure.

## 4. Conclusions

In this study, we found that hydrophobic amino acid residues (except for tyrosine) tend to be enriched in widened DNA minor grooves. We suggest that this phenomenon is not protein family-specific and features universally in protein–DNA complexes. Multiple interaction modes between the DNA helix and the minor groove-binding protein are involved. The sequence of the contacted DNA minor groove locus appears to be one of the groove width-determining factors. However, a mechanism explaining the enrichment of hydrophobic amino acid residues in wide minor grooves is yet to be described.

## Figures and Tables

**Table 1:** Differences between the amino acid frequency distributions found in narrow, standard, and wide minor grooves measured using relative entropy.

Distribution	DS1 Dataset Entropy [bit]	DS2 Dataset Entropy [bit]
Narrow to standard minor groove	0.58	0.61
Wide to standard minor groove	0.48	0.40
Wide to narrow minor groove	2.67	2.57

**Table 2:** Secondary structure preferences of hydrophobic amino acids indicated by numbers of observations.

		DS1 Dataset		DS2 Dataset	
	**Secondary**	**Count in**	**Count in**	**Count in**	**Count in**
**Residue**	**Structure**	**Standard min. g.**	**Wide min. g.**	**Standard min. g.**	**Wide min. g.**
ALA	α-helix	8	26	8	14
	β-strand	0	10	0	9
	others	13	5	12	5
	unassigned	12	9	12	8
GLY	bend	28	6	27	6
	turn	22	7	22	7
	β-strand	0	19	0	10
	others	13	7	13	4
	unassigned	19	6	19	5
ILE	β-strand	2	18	2	15
	α-helix	4	3	4	1
	others	2	2	2	3
	unassigned	5	1	5	0
LEU	α-helix	4	14	4	6
	β-strand	1	8	1	2
	others	4	3	4	1
	unassigned	2	1	2	0
PHE	3-10-helix	8	2	8	2
	α-helix	2	15	2	6
	others	5	6	4	2
	unassigned	4	12	4	4
TYR	α-helix	4	7	4	4
	others	9	2	9	2
	unassigned	10	1	10	1
VAL	β-strand	1	17	1	5
	α-helix	3	7	3	5
	others	3	2	3	2
	unassigned	7	2	7	1

**Table 3:** The numbers of residues for all amino acid types in the DS1 and DS2 datasets.

Amino Acid	ALA	ARG	ASN	ASP	CYS	GLN	GLY	GLU	HIS	ILE
DS1	83	310	69	27	2	45	131	27	40	37
DS2	68	294	53	27	1	45	116	21	38	32
**Amino Acid**	**LEU**	**LYS**	**MET**	**PHE**	**PRO**	**SER**	**THR**	**TRP**	**TYR**	**VAL**
DS1	38	117	26	54	56	72	62	15	41	42
DS2	21	111	16	36	45	64	52	14	37	27

## Supplementary Materials

The following are available online at https://www.mdpi.com/1422-0067/21/11/3986/s1: Figure S1: (**a**) Portion of amino acid residues that were assigned a hydrogen bonding interaction using the SNAP program. (**b**) Portion of amino acid residues assigned a van der Waals interaction. Table S1: Results of the one-sided Mann–Whitney *U* test for datasets DS1 and DS2. Figure S2: DNA-contacting amino acid preferences for dinucleotide step sequences. Table S2: Populations of hydrogen bonding interactions between the main chain atoms of amino acids and various moieties of the nucleotides in the three DNA minor groove width categories. Figure S3: DNA-contacting amino acid preferences for dinucleotide step sequence groups.
