# Supplementary material for: Hydrophobic Amino Acids as Universal Elements of Protein-Induced DNA Structure Deformation
Source: Int J Mol Sci. 2020 Jun 2;21(11):3986. doi: 10.3390/ijms21113986 (PMC7312683; doi:10.3390/ijms21113986)

Supplementary materials

Hydrophobic amino acids as universal elements of protein-induced DNA structure deformation

Kateřina Faltejsková David Jakubec and Jiří Vondrášek *

May 26, 2020

*To whom correspondence should be addressed. Tel: +420 220183267 ; Email: jiri.vondrasek@uochb.cas.cz

Table S1: Results of one-sided Mann–Whitney U test for datasets DS1 and DS2.

	DS1		DS2	
Amino acid	count	p-value	count	p-value
ALA	83	$7 \cdot 10^{-11}$	68	$3 \cdot 10^{-10}$
ASN	69	0.02	52	0.26
ASP	27	0.11	27	$5 \cdot 10^{-3}$
CYS	2	0.51	1	0.94
GLU	27	$2 \cdot 10^{-3}$	21	0.01
GLN	45	0.82	43	0.56
GLY	131	$6 \cdot 10^{-5}$	116	$8 \cdot 10^{-6}$
HIS	40	0.92	38	0.64
ILE	37	$5 \cdot 10^{-7}$	31	$7 \cdot 10^{-7}$
LEU	38	$2 \cdot 10^{-9}$	21	$3 \cdot 10^{-4}$
LYS	117	1	110	1
MET	83	$3 \cdot 10^{-4}$	15	0.09
PHE	54	$1 \cdot 10^{-10}$	35	$9 \cdot 10^{-7}$
PRO	56	0.23	45	0.27
SER	72	0.04	60	0.09
THR	62	0.03	49	0.29
TRP	15	0.58	14	0.28
TYR	41	0.84	37	0.76
VAL	42	$1 \cdot 10^{-10}$	27	$3 \cdot 10^{-6}$

Figure S1: **a)** Portions of amino acids that were assigned a hydrogen bond (calculated from all amino acids that were assigned an interaction by the SNAP program from the 3DNA suite). **b)** Portions of amino acids assigned a van der Waals interaction. If the amino acid does not occur in a given category, it is indicated by a dash.

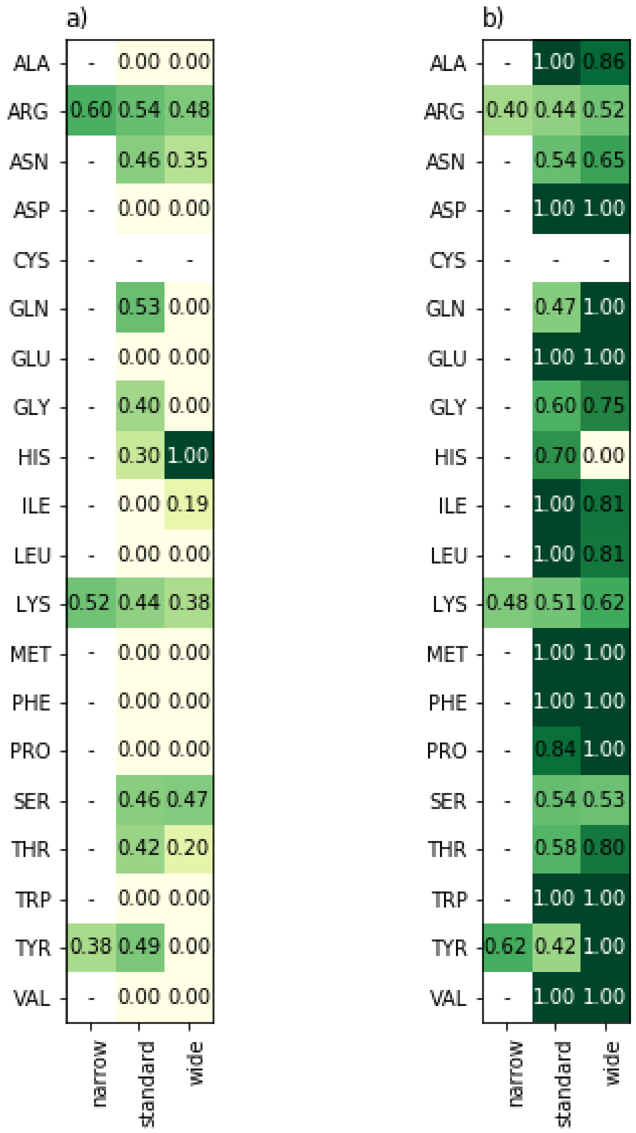

	aa	H-bond backbone -- base, narrow	H-bond backbone -- base, std	H-bond backbone -- base, wide	H-bond backbone -- phosphate, narrow	H-bond backbone -- phosphate, std	H-bond backbone -- phosphate, wide	H-bond backbone -- sugar, narrow	H-bond backbone -- sugar, std	H-bond backbone -- sugar, wide
0	ALA	0	3	4	0	2	0	0	0	0
1	ARG	5	14	3	20	26	0	2	3	1
2	ASN	0	2	0	1	2	0	0	0	0
3	ASP	0	0	0	0	2	0	0	0	0
4	GLN	0	3	1	0	4	0	0	1	0
5	GLY	4	50	4	1	0	1	2	15	0
6	HIS	0	0	0	0	1	0	0	0	0
7	ILE	0	2	3	0	2	0	0	0	0
8	LEU	0	1	1	0	1	0	0	0	0
9	LYS	2	2	5	2	18	0	0	2	1
10	MET	0	1	1	0	1	0	0	0	0
11	PHE	0	3	3	0	0	0	0	0	0
12	PRO	1	1	1	0	0	0	1	0	0
13	SER	0	2	1	1	2	1	0	3	0
14	THR	0	0	0	1	2	0	0	0	0
15	TRP	0	0	0	3	4	0	0	0	0
16	TYR	0	0	0	3	5	0	0	0	0
17	VAL	0	0	2	0	0	0	0	1	0

Table S2. Populations of hydrogen bonding interactions between the main chain atoms of amino acids and various moieties of the nucleotides in the three DNA minor groove width categories; DS1 dataset.

Figure S2: DNA-contacting amino acids preferences for dinucleotide step sequences.

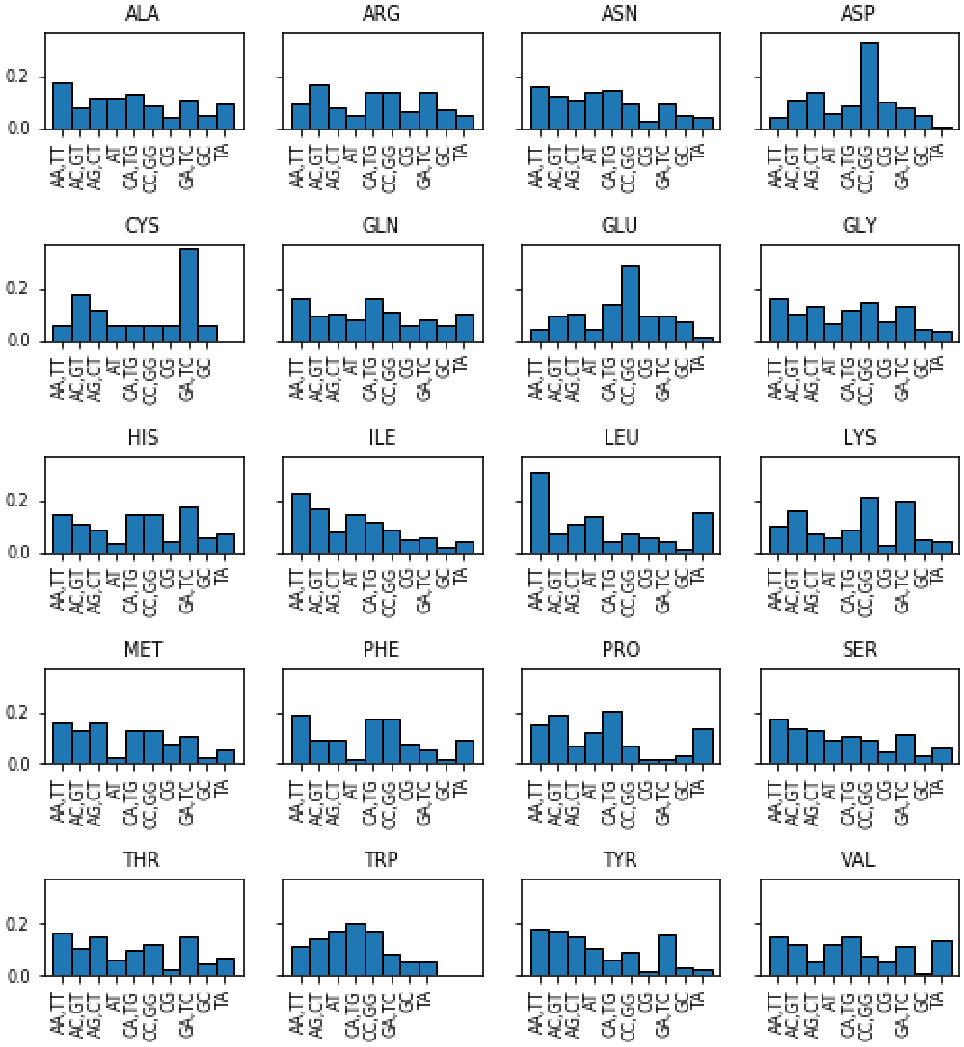

Figure S3: DNA-contacting amino acids preferences for dinucleotide step sequence groups. Sequences consisting only of A and T bases are classified into ”AT” group, similarly sequences consisting only of C and G bases. All sequences that do not belong to ”AT” or ”CG” group are classified into ”mix” group.

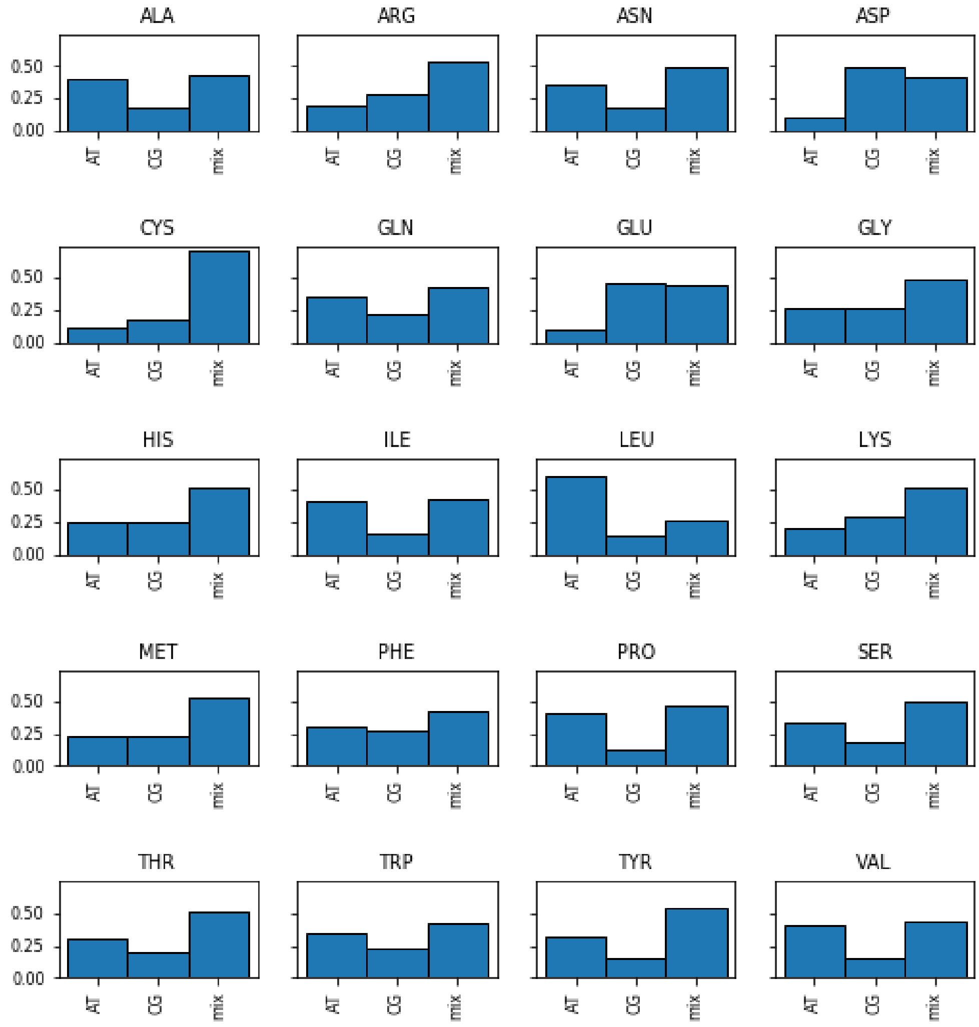

Supplement: Supplementary file 1 [file ijms-21-03986-s001.pdf]
